# Citrinin Exposure Induced Testicular Damage and Spermatogenesis Disorder by Triggering Endoplasmic Reticulum Stress

**DOI:** 10.3390/foods12081616

**Published:** 2023-04-11

**Authors:** Jing Wu, You Wu, Hui Fan, Chenglin Yang, Mengran Yang, Xiangyi Kong, Can Ning, Siqi Wang, Wenguang Xiao, Naidong Wang, Jine Yi, Zhihang Yuan

**Affiliations:** 1 Colleges of Veterinary Medicine, Hunan Agricultural University, Changsha 410128, China; 2 Hunan Engineering Research Center of Livestock and Poultry Health Care, Hunan Agricultural University, Changsha 410128, China; 3 Hunan Provincial Key Laboratory of Protein Engineering in Animal Vaccines, Laboratory of Functional Proteomics, Research Center of Reverse Vaccinology, Changsha 410128, China

**Keywords:** Citrinin, testicular damage, reproductive disorders, endoplasmic reticulum stress, oxidative stress, apoptosis

## Abstract

Damage to the reproductive system is the key factor leading to male infertility. Citrinin (CTN) is produced by Penicillium and Aspergillus in nature, and is definitely found in food and animal feed. Studies have revealed that CTN can cause damage to male reproductive organs and reduce fertility, but the mechanism of toxicity has not been revealed. In the present study, male Kunming mice were given different doses of CTN (0, 1.25, 5 or 20 mg/kg BW) by intragastric administration. The results demonstrated that CTN exposure caused disorder of androgen, a decline in sperm quality, and histopathological damage of testis. The inhibition of the expression of ZO-1, claudin-1 and occludin suggests that the blood-testis barrier (BTB) was damaged. Simultaneously, CTN inhibited the activity of antioxidant enzymes such as CAT and SOD, and promoted the production of MDA and ROS, resulting in oxidative damage of testis. Additionally, apoptotic cells were detected and the ratio of Bax/Bcl-2 was increased. Not only that, CTN activated the expression of endoplasmic reticulum stress (ERS)-related proteins IRE1, ATF6, CHOP, and GRP78. Interestingly, 4-Phenylbutyric Acid (4-PBA, an ERS inhibitor) treatment blocked the adverse effects of CTN exposure on male reproduction. In short, the findings suggested that CTN exposure can cause damage to mouse testis tissue, in which ERS exhibited an important regulatory role.

## 1. Introduction

In recent years, global warming and frequent rainfall in some areas have provided a suitable environment for mold growth, which accelerates the production of mycotoxins. Mycotoxins are diverse and widely distributed, polluting food and animal feed and causing acute or chronic toxicity to humans and animals [1]. Citrinin (CTN) can be found in cereals, fruits, vegetables, and red kojic rice, as well as in the commonly used food colorant red-pigmented *Monascus* [2]. A review of the global occurrence of CTN in foods found that CTN appears widely and seriously, especially in wheat flour, red yeast rice, and food additives (the range is between 1000 µg/kg and 121,097 µg/kg) [3]. This is far beyond the maximum safe dose published by the European Union, China, and Japan in the corresponding range. (The limit in Japan is 0.2 μg/g, and the limit in the EU is 2 μg/g) The increased occurrence of CTN contamination has greatly increased the probability of human and animal contact and subsequent risk of toxicity.

In recent years, several studies have reported that CTN exposure can cause multiple types of organ and system damage, which can manifest as hepatotoxicity, nephrotoxicity, or reproductive toxicity [4,5,6,7]. Worldwide, reproductive system damage is a direct cause of infertility. The World Health Organization (WHO) reported that 72.4 million couples worldwide suffer from infertility, and nearly half of the causes may be related to diseases of the male reproductive system [8]. The factors that cause reproductive dysfunction are diverse and complex but the role of mycotoxin exposure cannot be ignored [9]. The threat of CTN exposure to reproductive health is increasingly recognized. Studies have reported the effect of CTN on female reproduction, indicating that CTN can have adverse effects on ovaries and oocytes [5,10]. However, research on how CTN affects the male reproductive system is comparatively rare. In a previous study, after intraperitoneal injection of CTN (6.25 mg/kg body weight) in male mice, the relative weights of testes, epididymis, seminal vesicles, and preputial glands were significantly increased; the rate of sperm deformity was increased; and sperm motility was decreased [11], but its mechanism of action was not clarified. Subsequently, in vitro research further proved that CTN inhibited Sertoli cell proliferation and induced apoptosis [12]. However, the above studies do not fully explain the mechanism of CTN-induced male reproductive toxicity.

Previous studies have reported that CTN toxicity is primarily mediated via oxidative stress and apoptosis [4]. Similarly, our previous study confirmed that CTN triggers oxidative stress and apoptosis in hepatocytes in vivo and in vitro, and this is regulated by endoplasmic reticulum stress (ERS) [6,13]. In the context of enterotoxicity, it has also been reported that CTN causes ERS-induced apoptosis in intestinal cells [14]. Therefore, we hypothesized that ERS would also plays an important role in CTN-induced male reproductive toxicity.

In this study, we used a CTN-induced reproductive injury model in male mice to explore the toxic effects of CTN on the testes and the mechanism of ERS in testicular injury and spermatogenesis impairment. This has clinical significance for the prevention or treatment of reproductive disorders caused by CTN.

## 2. Materials and Methods

### 2.1. Ethical Statement

This study complies with pertinent provisions of the Hunan Agricultural University Experimental Animal Ethics and Use Committee’s bylaws, and the Animal Health Committee has approved it. The China Animal Health and Use Guidelines were followed in all animal experimentation. Ethical approval number No. 43322087.

### 2.2. Chemicals and Reagents

CTN was bought from Pribolab Biological Technical Co., Ltd., (Qingdao, China). MedChemExpress (MCE) provided the 4-PBA. Glutathione (GSH) (A006-2-1), malondialdehyde (MDA) (A003-1-2), superoxide dismutase (SOD) (A001-3-2), catalase (CAT) (A007-1-1), and BCA assay kit (A045-4-2) were purchased from Nanjing Jiancheng Institute of Biological Engineering, and immunofluorescence-related dyes were obtained from Servicebio (Hubei, China). Primary antibodies against Bcl-2 and Bax were purchased from Cell Signaling Technology (Danvers, MA, USA). Primary antibodies against β-actin(81115-1-RR), ATF6(24169-1-AP), IRE1(27528-1-AP), GRP78(11587-1-AP), and CHOP (15204-1-AP) were obtained from Proteintech Group, Inc. (Chicago, IL, USA). The primary and secondary antibodies against occludin, cluadin-1, and ZO-1 were all from Aifang Biotechnology (Hunan, China).

### 2.3. Animals and Treatment

Twenty male Kunming mice aged 7 weeks were chosen and bought from SJA (Hunan, China). They were divided into four groups as follows (*n* = 5) after one week of acclimation feeding: a blank group, and low- (CTN 1.25 mg/kg b.w), medium- (CTN 5 mg/kg b.w.), and high- (CTN 20 mg/kg b.w.) dose groups. 10 mg CTN was dissolved in 1 mL anhydrous ethanol and then further diluted with double-distilled water to ensure the solvent concentration was 2% ethanol. The low-, medium-, and high-dose groups of CTN were intragastrically administered CTN suspension at the prescribed dose, while the blank group received 2 percent ethanol, once a day for 14 days.

In order to explore the specific mechanism of CTN-induced testicular injury in mice, we conducted a further test. After acclimation for one week, twenty male Kunming mice were split into four groups (*n* = 5): control group, CTN group, 4-PBA group, and CTN+4-PBA group. 4-PBA (240 mg/kg body weight) was injected intraperitoneally 30 min before CTN administration; control and 4-PBA groups were administered a 2% ethanol solution orally, and the remaining groups were administered CTN suspension diluted with 2% ethanol solution orally, once a day for 14 days. (Systemic reactions occurred in mice given 20 mg/kg b.w. of CTN during the pilot phase, possibly as a result of an excessively toxic dose; 5 mg/kg b.w. was chosen as the final dose.) During the experiment, the mice had unrestricted access to food and water and were kept in light and dark for 12 h per day. Mice were fasted for 12 h prior to dissection, after which blood was drawn from the eyes and serum was collected. Testes and the epididymis were then collected for use in later experiments.

### 2.4. Calculation of the Testicular Index

Before dissection, we recorded the live body weight of each mouse. After orchiectomy, the testes in each group were evaluated and weighed. The testicular organ index was then computed using the formula below.

Testicular organ index (%) = testicular organ weight (mg)/mouse live weigh (g) × 100%

### 2.5. Histopathological Examination

We cut the testicular tissue into 5 µm thick sections using a microtome to prepare paraffin-embedded tissue sections. They were dewaxed in xylene and rehydrated through a series of graded alcohols (100%, 90%, 70%, 50%). They were then stained for 5 min with hematoxylin, a basic dye that binds to acidic components such as DNA and RNA. After rinsing with tap water, the sections were immersed in 1% hydrochloric acid and 95% ethanol acidified with ammonia water to distinguish cell nuclei. After 3–5 min of eosin staining (the combination of eosin and collagen, cytoplasmic protein, and other basic components will stain pink or red), we removed excess paint under running water, then dehydrated gradually with alcohol. Finally, we let it dry, sealed it, and inspected it.

### 2.6. Transmission Electron Microscope Examination

Transmission electron microscopy (TEM) is a powerful tool for visualizing internal structures of cells and other biological samples at high resolution. The following is a basic method for TEM examination of biological specimens:

Within 1–3 min of opening the abdominal cavity, samples were obtained, and testicular tissues measuring 2 mm by 2 mm were soaked in glutaraldehyde fixative and preserved for 24 h. We embedded the samples in resin and polymerized the resin following manufacturer instructions. Resin embedding provides structural support and allows for thin sectioning of the samples. We stained the sections with heavy metal stains such as uranyl acetate and lead citrate to increase the contrast of the sample regions, before they were examined and photographed using a transmission electron microscope (H-7500, Hitachi, Tokyo, Japan). We examined the stained sections of the samples under the TEM, adjusting the focus, magnification, and other settings to achieve optimal image quality. We captured images of the samples for further analysis.

### 2.7. Sperm Viability Test

The tail of the epididymis on one side of the mouse was removed. We added 1 mL of warm saline at 37 °C, and the epididymis was cut on the thermostat plate to release the sperm. We then used a pipette gun to suck 200 μL of semen into a sperm counting plate, and the viability of the sperm was assessed using an automatic sperm quality analyser (ML-80III, Mylan, Nanning, China).

### 2.8. Testosterone Concentration Measurement

Serum testosterone levels were detected using a Testosterone Enzyme-Linked ImmunoSorbent Assay Kit (AF2569, Hunan, Aifang Bio) and procedures were performed according to the manufacturer’s instructions. Briefly, the serum was incubated with the enzyme conjugate solution for 2 h at room temperature away from light in the ELISA plate. After washing the plate three times, the tetramethylbenzidine chromogen (TMB) was added and incubated for 20 min at room temperature in the dark. Then the stop solution was added and the absorbance was measured at 450 nm using a microplate reader.

### 2.9. Reactive Oxygen Species (ROS) Assay

Testicular tissue was frozen with liquid nitrogen; this quick-freezing technique helps to preserve the cellular structure and minimize ROS generation during sample manipulation. We cut the frozen samples into thin sections (5–10 µm) using a cryostat or similar device, and washed the sections 2–3 times in phosphate-buffered saline (PBS) to remove excess fixative. After this, we incubated the sections in a fluorescent ROS probe (such as DCFDA or DHE) for 30–60 min at room temperature. These probes detect reactive oxygen species by undergoing fluorescence changes in response to ROS levels. We mounted the sections with a mounting medium containing DAPI or other nuclear dye to visualize cell or tissue morphology. We observed the sections using a fluorescence microscope, using appropriate excitation and emission filters for the ROS probe and nuclear dye, respectively. We analysed the resulting images to quantify ROS levels in the testicular tissues.

### 2.10. Oxidation-Antioxidant Index Detection

The testicular tissue of mice frozen at −80 °C was taken as 0.1 g/each, thawed, diluted 10 times with saline for tissue homogenization, centrifuged at 2500 r/min for 10 min, and then the supernatant was collected for further analysis. The protein concentration was measured by BCA(A045-4-2) kit. The levels of GSH(A006-2-1), MDA(A003-1-2), CAT(A007-1-1) and SOD(A001-3-2) in testis tissues were detected and calculated using kit instructions from Nanjing Jian Cheng Institute of Biological Engineering (Nanjing, China).

### 2.11. TUNEL Assay

Testicular tissue sections embedded in paraffin underwent strict pretreatment in accordance with the TUNEL Apoptosis Assay Kit’s instructions. After incubated with TdT enzyme reaction solution at 37 °C for 60 min, sections were treated with DAB (50–100 μL) working solution for 5–30 min at room temperature and then treated with an antifluorescent antibody for 30 min in the dark. An inverted fluorescence microscope was utilized to observe apoptotic cells. After the required images were collected, Image J was used to quantify the fluorescence intensity.

### 2.12. Western Blotting Assay

Testicular tissue of mice frozen at −80 °C was taken as 0.1 g/each, thawed, added to nine times the RIPA Lysis Buffer (R0010; Solarbio, Metro Manila, Philippines), homogenized on ice, and centrifuged at 2500 r/min for 10 min to extract the supernatant. The total amount of extracted protein was quantified using the BCA(A045-4-2; njjcBio) protein assay kit. For western blotting, equal protein samples (30 µg) were loaded and separated using 8–12% SDS-PAGE gel, and then transferred onto a nitrocellulose membrane (IPVH00010; Millipore, Burlington, MA, USA). Subsequently, the membrane was blocked with 5% skim milk dissolved in TBST for 2 h at room temperature and incubated overnight at 4 °C with the primary antibodies. After washing with TBST and incubation for 1.5 h at room temperature with the secondary antibody, the probes were detected using electrochemiluminescence (ECL) reagents, exposed using ChemiDoc XRS (Bio-Rad, Hercules, CA, USA). The blot grey value was quantified using Image J.

### 2.13. Immunofluorescence Staining

Testicular paraffin sections were permeabilized treated with 0.25% (*v*/*v*) Triton X-100 for 20 min at 37 °C. The sections were blocked with 3% BSA 30 min and appropriate amount of ZO-1 primary antibody was added for incubation at 4 °C overnight. The following day, the samples were washed with PBS and incubated with secondary antibodies at room temperature for 2 h in the dark, and then underwent nuclear staining using 4′,6-diamidino-2-phenylindole (DAPI). Images were acquired with a Nikon fluorescence microscope, and fluorescence intensity and density analysis were performed using Image J.

### 2.14. Statistical Analysis

All data were presented as mean ± SEM and analysed using SPSS Statistics software (version 25.0, IBM Agency, Armonk, NY, USA). Differences between experimental groups were assessed by one-way ANOVA. Values of *p* < 0.05 were considered statistically significant, and values of *p* < 0.01 were considered extremely significant.

## 3. Results

### 3.1. The Effects of CTN on Testicular Damage

**Figure 1:**
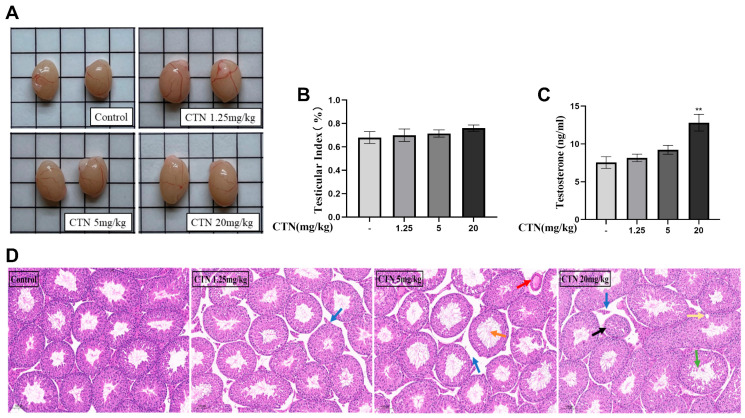
Effects of CTN on testicular damage in mice. (**A**) The appearance of mice testes; (**B**) The testicular index; (**C**) Serum testosterone concentration; (**D**) Histopathological analysis in testis tissue. (Blue: enlarged intercellular matrix; orange: increased luminal diameter; red: vascular congestion and dilatation; black: luminal obstruction; yellow: vacuolation of germinal tubules; green: luminal cell detachment; scale bar: 100 μm). The above data are expressed as SEM ± mean, compared with the control group, ** *p* < 0.01.

As seen in Figure 1A,B, CTN appeared to cause testicular swelling and increased testis index, but the differences were not statistically significant. However, Figure 1C demonstrated that CTN elevated serum testosterone concentrations in a dose-dependent way. It was significantly upregulated (*p* < 0.01) in the 20 mg/kg CTN group. Moreover, the histopathological examination revealed that CTN induced abnormal morphology of the spermatogenic tubules, including loose and disorganized arrangement, increased lumen diameter and vacuolization, lumen cell abscission and blockage, and decrease in spermatogonia and mature sperm (Figure 1D).

### 3.2. The Effects of CTN on Spermatogenesis

**Figure 2:**
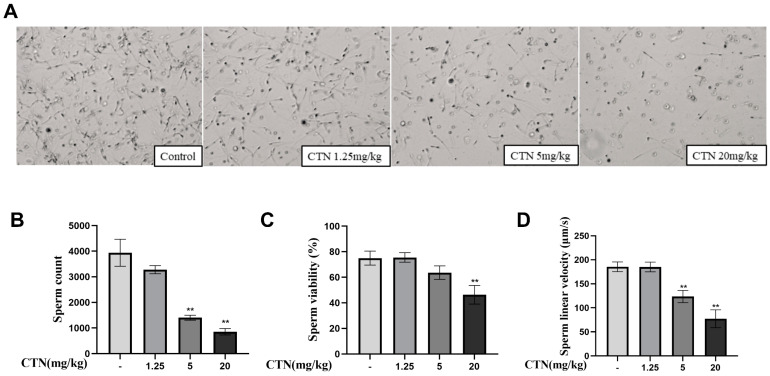
Effects of CTN on sperm quality in mice. (**A**) sperm count and morphology; (**B**) Sperm count; (**C**) Sperm viability; (**D**) Sperm linear velocity. The above data are expressed as SEM ± mean, compared with the control group, ** *p* < 0.01.

The results are shown in Figure 2. After 5 mg/kg and 20 mg/kg CTN exposure, the sperm count (Figure 2A,B) of mice was significantly reduced (*p* < 0.01), and sperm motility (Figure 2C) decreased with increasing CTN concentration in each experimental group, with highly significant differences at 20 mg/kg CTN treatment compared to the control group. In addition, the sperm linear velocity (Figure 2D) was also significantly inhibited (*p* < 0.01).

### 3.3. The Effects of CTN on Testicular Oxidative Damage

**Figure 3:**
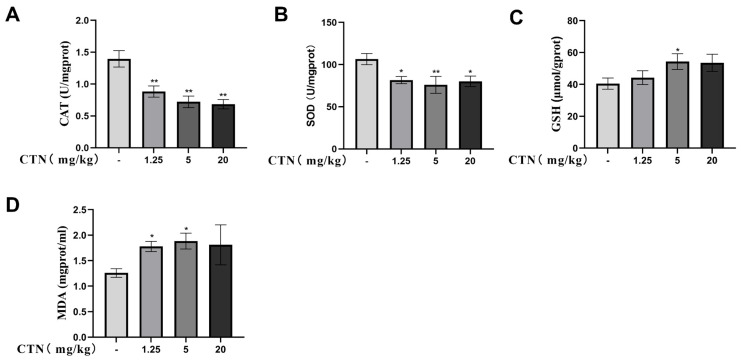

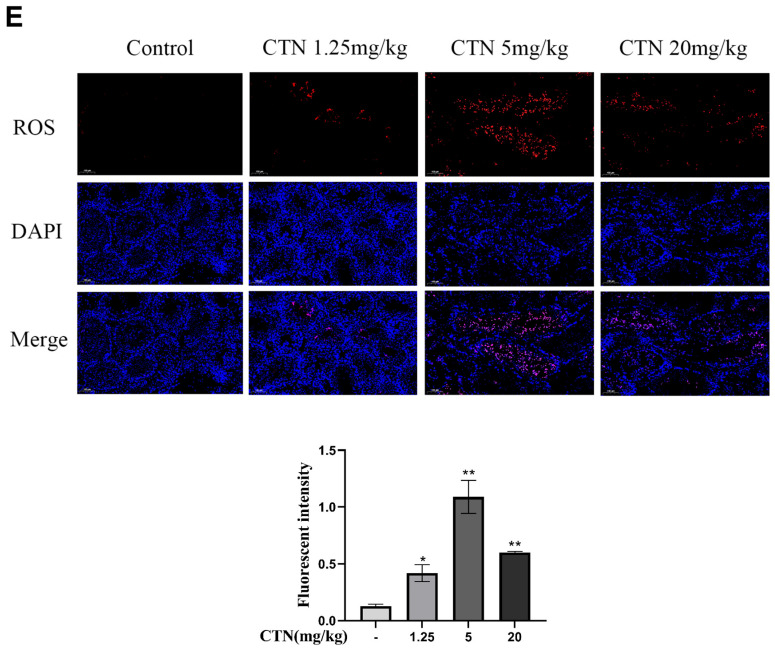
Effects of CTN on testicular oxidative damage. (**A**) CAT activity; (**B**) SOD activity; (**C**) GSH content; (**D**) MDA content; (**E**) ROS content in testicular tissue; scale bar in figure: 100 μm. The above data are expressed as SEM ± mean, compared with the control group, * *p* < 0.05, ** *p* < 0.01.

The effects of CTN exposure on oxidative stress in mouse testes were then examined. As seen in Figure 3, CTN significantly inhibited (*p* < 0.01) the activities of antioxidant enzymes CAT (Figure 3A) and SOD (Figure 3B) and increased (*p* > 0.05) the content of both GSH (Figure 3C) and MDA (Figure 3D). Similarly, fluorescence microscopy showed that the ROS content (Figure 3E) in testis tissue was significantly increased (*p* < 0.01) after CTN exposure.

### 3.4. The Effects of CTN on Apoptosis of Testicle Cells

**Figure 4:**
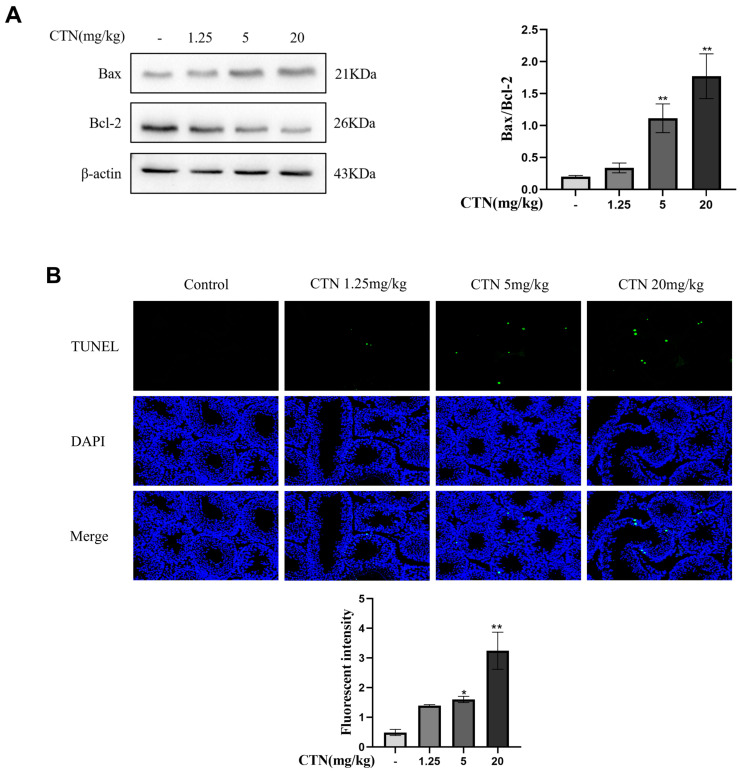
Effects of CTN on apoptosis of testicle cells in mice. (**A**) Bax and Bcl-2 protein expression levels; (**B**) TUNEL assay for testicular apoptosis (scale bar: 100 μm); the above data are expressed as SEM ± mean, compared with the control group, * *p* < 0.05, ** *p* < 0.01.

In the present study, the effects of CTN exposure on apoptosis in mouse testis tissue was examined, and the results are shown in Figure 4. The changes in the ratio of Bax/Bcl-2 (Figure 4A), a key index regulating apoptosis, were analysed by Western Blot, and the results showed that the ratio was significantly upregulated (*p* < 0.01) after CTN exposure. Fluorescence microscopy observed that the intensity of green fluorescence (apoptotic cells) in testicular tissue was significantly increased (*p* < 0.01) after exposure to CTN (Figure 4B).

### 3.5. Effects of 4-PBA on Testicular ERS and Ultrastructural Changes after CTN Exposure

**Figure 5:**
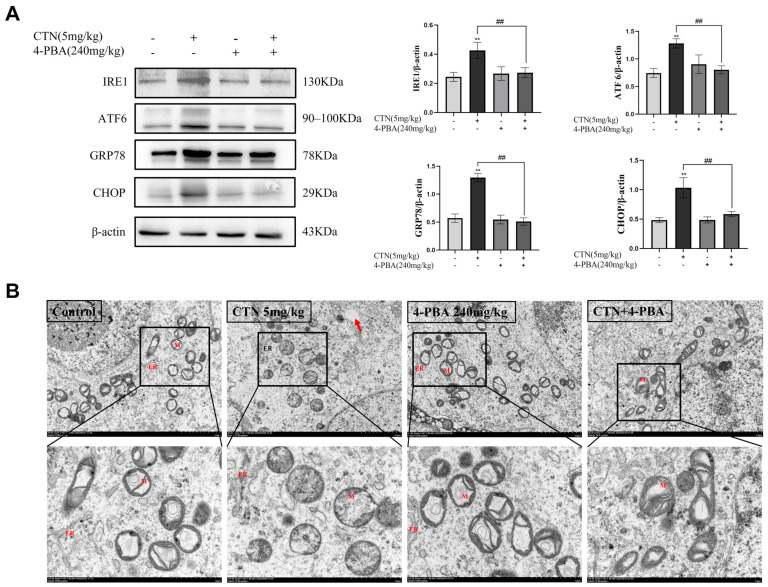
The effects of 4-PBA testicular ERS and ultrastructural changes after CTN exposure. (**A**) IRE1, ATF6, GRP78, CHOP protein expression levels; (**B**) Electron microscopic ultrastructural examination of the testis. (N: nucleus; M mitochondria; →: vacuole-like structures; ER: endoplasmic reticulum). The above data are expressed as SEM ± mean, ** *p* < 0.01, compared with the control group, ## *p* < 0.01, compared with the CTN group.

A small-molecule fatty acid, 4-PBA works as an endoplasmic reticulum stress-specific blocker to stop the activation of endoplasmic reticulum stress by reversing the misdisplacement or misaggregation of protein molecules and assisting them in establishing a normal spatial structure. We used this substance in the current investigation to look into the toxicity of CTN in male mice following 4-PBA pretreatment, by analysis of ERS-related proteins in testis tissue after exposure to CTN (5 mg/kg). The results are shown in Figure 5A. Compared with the CTN group, the expression levels of ERS regulatory proteins IRE1, ATF6, GRP78 and CHOP were significantly downregulated (*p* < 0.01) after 4-PBA treatment. This indicated that ERS activation was inhibited after exposure to CTN. In addition, ultrastructural examinations showed that CTN induced a large number of vacuoles in cytoplasm, mitochondrial swelling and cristae disruption, and ER dilation and ribosome particles shedding in the testes of mice, which were reversed by pretreatment with 4-PBA (Figure 5B).

### 3.6. Effects of 4-PBA on Testis Cells Apoptosis after CTN Exposure

**Figure 6:**
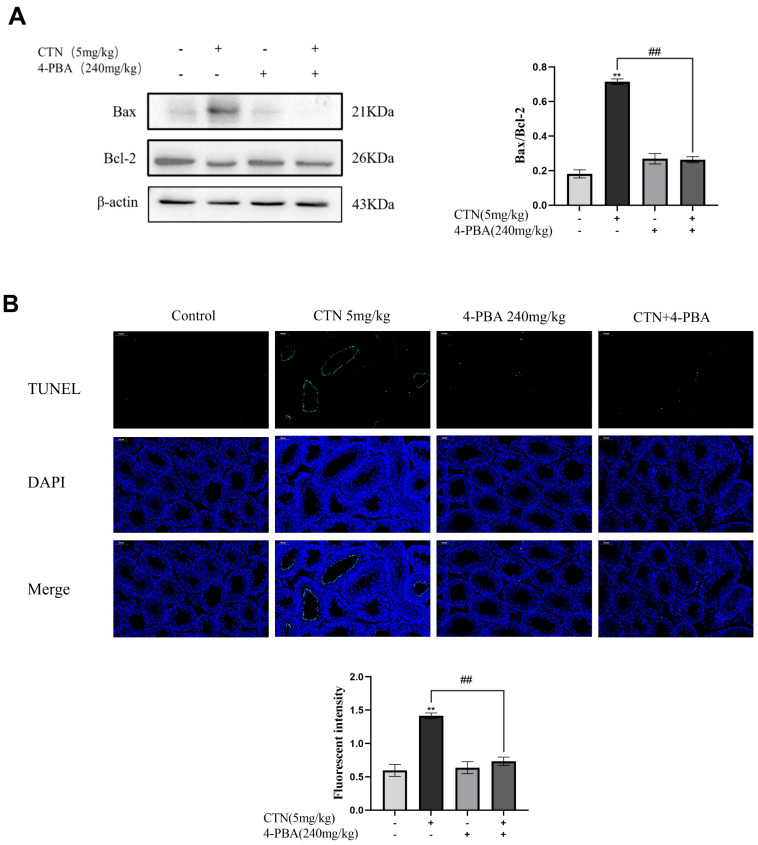
The effects of 4-PBA on CTN-induced apoptosis in testicular cells. (**A**) Bax, Bcl-2 protein expression levels; (**B**) TUNEL assay for testicular apoptosis. The above data are expressed as SEM ± mean, ** *p* < 0.01, compared with the control group, ## *p* < 0.01, compared with the CTN group.

ERS is one of the endogenous pathways that cause apoptosis. Compared with the CTN group, 4-PBA pretreatment significantly decreased (*p* < 0.01) the ratio of Bax/Bcl-2 (Figure 6A), and significantly reduced (*p* < 0.01) the apoptosis of testicular cells caused by CTN exposure (Figure 6B).

### 3.7. Effects of 4-PBA on Testicular Oxidative Damage after CTN Exposure

**Figure 7:**
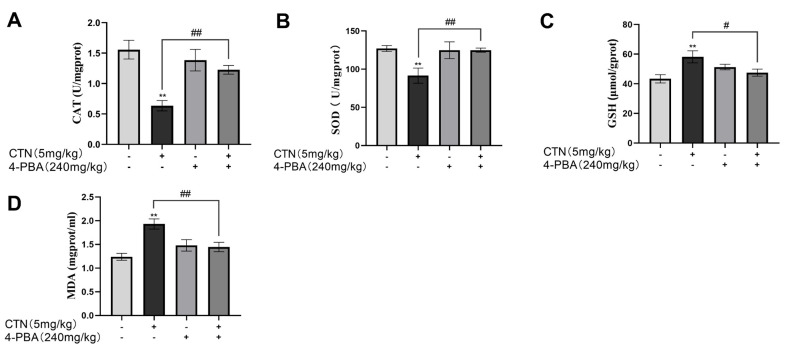

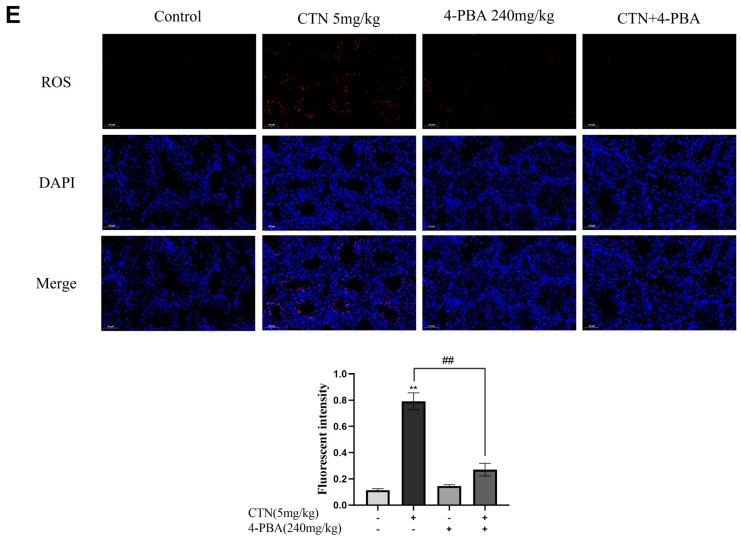
The effects of 4-PBA on CTN-induced testicular oxidative damage in mice. (**A**) CAT activity; (**B**) SOD activity; (**C**) GSH content; (**D**) MDA content; (**E**) ROS content in testicular tissue; scale bar in figure: 100 μm. The above data are expressed as SEM ± mean, ** *p* < 0.01, compared with the control group, # *p* < 0.01, ## *p* < 0.01, compared with the CTN group.

It has been confirmed that CTN induced oxidative damage in mice testes. After 4-PBA pretreatment, it was found that compared with the CTN group, the activities of antioxidant enzymes CAT and SOD were increased (Figure 7A,B), the content of GSH (Figure 7C) returned to the normal level, and the antioxidant capacity was enhanced. In addition, the levels of MDA and ROS were significantly reduced (Figure 7D,E, *p* < 0.01), which showed that CTN-induced testicular injury was alleviated.

### 3.8. Effects of 4-PBA on Blood Testis Barrier (BTB) Damage after CTN Exposure

**Figure 8:**
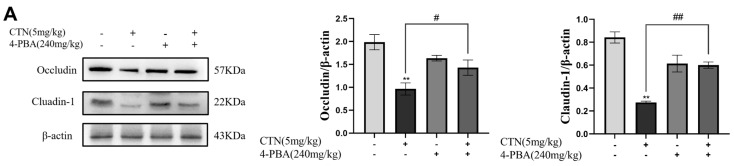

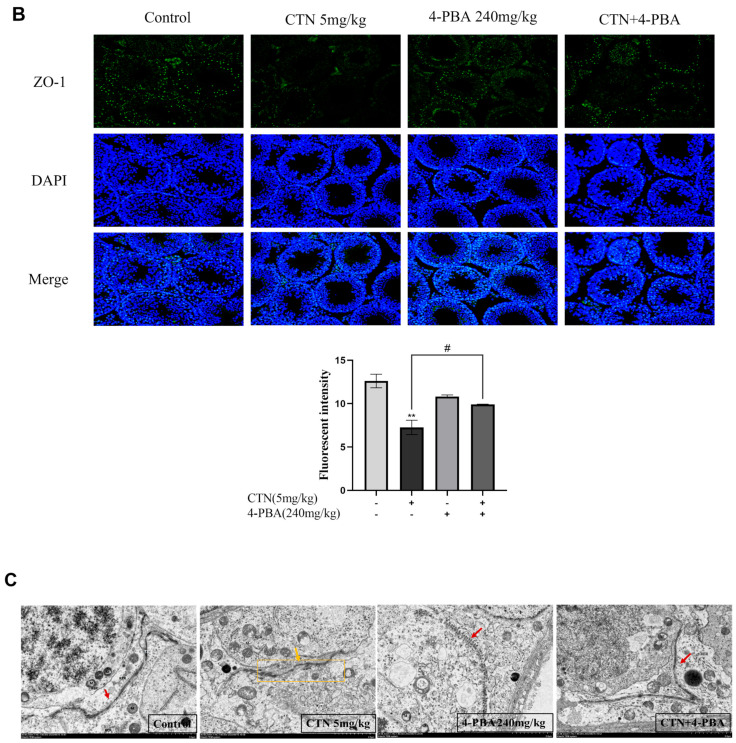
Effects of 4-PBA on CTN-induced BTB damage in mice. (**A**) Occludin and claudin-1 protein expression levels; (**B**) ZO-1 immunofluorescence; (**C**) Ultrastructural observation of testicular tissue (Red: TJ; yellow: TJ destruction; M: mitochondria; RER: endoplasmic reticulum; scale bar: 2 μm. The above data are expressed as SEM ± mean, ** *p* < 0.01, compared with the control group, # *p* < 0.05, ## *p* < 0.01, compared with the CTN group.

Compared with the control group, after exposure to CTN, the expression of tight junction proteins occludin and claudin-1 was significantly reduced (Figure 8A, *p* < 0.01), and the results of immunofluorescence detection showed that the fluorescence intensity of ZO-1 was significantly downregulated (Figure 8B, *p* < 0.01). In addition, the transmission electron microscopy results showed that the TJ indicated by the arrow in the CTN group showed blurred structure compared with the blank group, which indicated that the BTB-related structures in the testis were damaged (Figure 8C). The BTB damage caused by CTN was reversed after 4-PBA pretreatment.

### 3.9. Effects of 4-PBA on Testicular Damage after CTN Exposure

**Figure 9:**
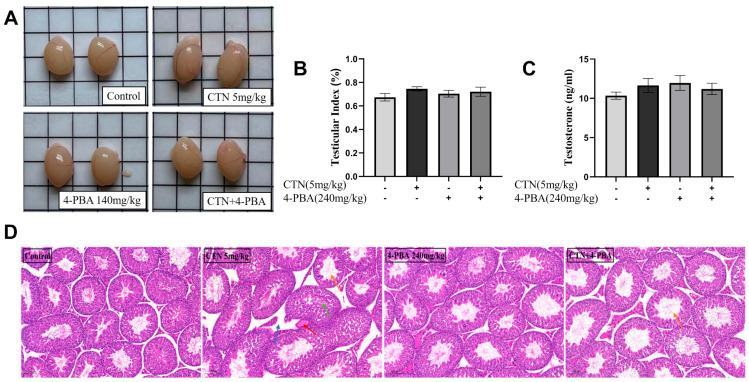
The effects of 4-PBA on testicular damage induced by CTN. (**A**) Morphological observation of testes; (**B**) Testicular index; (**C**) Serum testosterone concentration; (**D**) Assessment of histopathological damage. (Blue: enlarged intercellular matrix; orange: increased luminal diameter; red: congested and dilated blood vessels; green: detached luminal cells). The above data are expressed as SEM ± mean, compared with the CTN group, *p* < 0.05.

The results of testicular morphology and histopathology showed that 4-PBA pretreatment had no significant effect on testicular appearance or serum testosterone compared with CTN group (Figure 9A–C, *p* > 0.05). However, histopathological examination showed that the testicular tissue damage caused by CTN exposure was repaired after 4-PBA pretreatment (Figure 9D).

### 3.10. Effects of 4-PBA on Spermatogenesis Disorder after CTN Exposure

**Figure 10:**
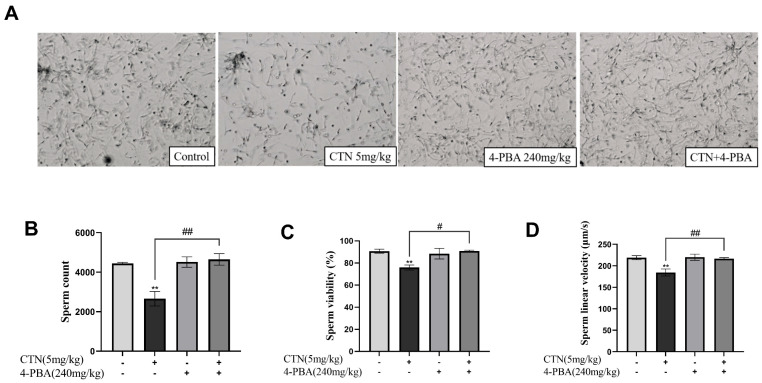
The effects of 4-PBA on CTN-induced spermatogenesis disorders in mice. (**A**) sperm count and morphology; (**B**) Sperm count; (**C**) Sperm viability; (**D**) Sperm linear velocity. The above data are expressed as SEM ± mean, ** *p* < 0.01, compared with the control group, # *p* < 0.05, ## *p* < 0.01, compared with the CTN group.

Sperm concentration, survival rate and linear velocity are important indicators to measure sperm quality. In this study, compared with the CTN group, 4-PBA pretreatment can significantly improve the sperm number and sperm linear velocity (Figure 10A,C, *p* < 0.01), but has no significant impact on sperm viability (Figure 10B, *p* > 0.05).

## 4. Discussion

Approximately 700 million tons of grain are wasted annually owing to the invasion of various molds despite the fact that the globe produces 4 billion tons of grain annually. Mycotoxin contamination not only reduces grain yield and quality, but also endangers human and animal health. As a result, mycotoxin contamination is now a well-acknowledged issue with food safety. One of the potentially dangerous poisons is CTN, which mainly enters the body through food and animal products, threatening human and animal health. CTN has been demonstrated to cause nephrotoxicity, hepatotoxicity, teratogenicity, and reproductive toxicity. Although a variety of toxic effects of CTN have been uncovered, the understanding of reproductive toxicity, especially the role and mechanism of testicular damage in males, is relatively rare. The testes are the most important reproductive organs for men, responsible for sperm production and androgen secretion. The main function of testosterone is to maintain secondary sexual characteristics and promote the development of sexual organs [15]. Damage to the testes by toxicants not only affects their development but also leads to infertility [16]. A previous study mentioned that continuous exposure to CTN for seven days resulted in increased testicular, epididymal, and seminal vesicle weights, as well as disturbances in spermatogenesis and testosterone secretion [11]. In addition, in vitro studies on Leydig cells also confirmed that CTN can inhibit testosterone synthesis and trigger cell apoptosis [17]. Similarly, in the present study, CTN exposure resulted in mild testicular enlargement and histopathological damage. Although some studies have shown that CTN exposure leads to elevated serum testosterone, our study found that serum testosterone concentrations were reduced in mice exposed to CTN. This may be related to androgen receptor inhibition or dysregulation of adrenal-derived androgen secretion [18]. Additionally, the reduced sperm count, motility, and linear velocity suggest that CTN can impair spermatogenesis. Collectively, CTN can cause testicular damage, impaired spermatogenesis, androgen secretion disorders, and other adverse effects which seriously threaten male reproductive health.

Testicular tissues or cells have their own antioxidant system, composed of antioxidant enzyme systems (SOD, CAT, etc.) and nonenzymatic systems (GSH, etc.) to maintain the normal operation of the reproductive system [19]. Excessive stimulation of adverse factors will lead to an imbalance between oxidant and antioxidant systems, resulting in oxidative damage affecting spermatogenesis, and even infertility [20,21]. The destiny of sperm is usually determined by reactive oxygen species (ROS) production and GSH depletion, and ROS accumulation accelerates lipid peroxidation, causing damage to sperm membrane structure and DNA [22]. Previous studies have shown that the induction of oxidative stress is an underlying mechanism of CTN toxicity [23,24]. However, compared with other tissues, testicular cells are more vulnerable to oxidative stress, due to their rapid cell division pace and high rate of mitochondrial oxygen consumption [25]. In the present study, it was confirmed that CTN induced oxidative stress in testes, which was reflected by the significant increase in ROS and MDA content, and the decrease in CAT and SOD enzyme activity, which seemed to be an important mechanism of CTN-induced testicular injury.

Oxidative stress is also involved in the induction of apoptosis [26]. Indeed, apoptosis was found to drive CTN-induced Sertoli and Leydig cell injury in mice and rats, respectively [12,17], and previous studies have demonstrated the relationship between CTN-induced apoptosis and oxidative stress [24] Therefore, CTN-induced testicular injury and spermatogenesis impairment may depend on germ cell apoptosis. TUNEL staining confirmed that CTN increased testicular cell apoptosis, suggesting that spermatogenesis impairment may be caused by apoptosis. Furthermore, CTN significantly increased the ratio of pro-apoptotic Bax/anti-apoptotic Bcl-2, a key measure of apoptosis occurrence [27], confirming the activation of germ cell apoptosis.

The endoplasmic reticulum (ER) is a key part of protein synthesis and processing, and its homeostatic imbalance leads to ERS. ERS is another endogenous apoptosis pathway besides the mitochondrial pathway [28]. It has been demonstrated that ERS, oxidative stress, and apoptosis are interrelated. Our previous studies confirmed that inhibition of ERS reduced the hepatotoxicity of CTN in vivo and in vitro, which was related to inhibition of oxidative stress and apoptosis [6,13]. Therefore, CTN-induced testicular injury and spermatogenesis impairment may be attributed to ERS. In this study, CTN exposure increased the expression of IRE1, ATF6, GRP78, and CHOP, and caused ER expansion, indicative of ERS. Furthermore, these changes were prevented by 4-PBA and these were accompanied by a reduction in testicular cell apoptosis and a decrease in the ratio of Bax/Bcl-2, indicating that CTN-induced apoptosis was mediated via ERS. Similarly, under 4-PBA pretreatment, the activity of antioxidant enzymes in the testes was significantly increased and the contents of ROS and MDA were decreased, confirming that ERS could mediate oxidative stress. Taken together, these results suggest that CTN toxicity in the male reproductive system may be mediated via ERS.

The BTB is an important defence line for the testes in resisting hazardous chemical invasion and maintaining normal physiological function [29]. Tight junctions are an essential component of the BTB, and the expression of related proteins such as occludin, claudin-1, and ZO-1 is required for barrier maintenance and material transit [30]. According to several research reports, mycotoxin-induced oxidative stress is the beginning mechanism of BTB damage, which eventually leads to testicular injury and spermatogenesis impairment by degrading BTB-related proteins and promoting Sertoli cell apoptosis [31,32,33,34]. As a result, the toxicity of CTN in the testes may be linked to BTB damage. CTN did, in fact, diminish the expression of occludin, claudin-1, and ZO-1 in this study, as well as demolish tight junctions between cells. A recent study showed that ERS can lead to BTB damage [35]. Similarly, this study found that 4-PBA counteracted the CTN-induced reduction in tight junction protein expression. In addition, testis damage and spermatogenesis impairment induced by CTN were also prevented.

## 5. Conclusions

As a common and unavoidable environmental toxin, CTN has a clear toxic effect on male reproductive system, which is reflected in pathological damage of testicular tissue and spermatogenesis disorder. It is mainly realized by inducing oxidative damage, cell apoptosis, and destruction of the BTB. Inhibition of ERS can alleviate the above adverse effects, which can be used as an important way to combat the reproductive toxicity of CTN. In conclusion, the results of this study provide a basis and new ideas for clarifying the reproductive toxicity mechanism of CTN and developing therapeutic drugs.

## Data Availability

The data that support the findings of this study are available from the corresponding author [zhyuan2016@hunau.edu.cn], upon reasonable request.
